# Cold-air outbreaks in the continental US: Connections with stratospheric variations

**DOI:** 10.1126/sciadv.adq9557

**Published:** 2025-07-11

**Authors:** Laurie Agel, Judah Cohen, Mathew Barlow, Karl Pfeiffer, Jennifer Francis, Chaim I. Garfinkel, Marlene Kretschmer

**Affiliations:** ^1^ Department of Environmental, Earth, and Atmospheric Sciences, University of Massachusetts Lowell, Lowell, MA 01854, USA.; ^2^ Atmospheric and Environmental Research Inc., Lexington, MA 02421, USA.; ^3^ Department of Civil and Environmental Engineering, Massachusetts Institute of Technology, Cambridge, MA 02139, USA.; ^4^ Atmospheric and Environmental Research, Hampton, VA 23666, USA.; ^5^ Woodwell Climate Research Center, Falmouth, MA 02540, USA.; ^6^ The Hebrew University of Jerusalem, Institute of Earth Sciences, Edmond J. Safra Campus, Jerusalem, Israel.; ^7^ Leipzig Institute for Meteorology, University of Leipzig, Leipzig, Germany.; ^8^ Department of Meteorology, University of Reading, Reading, UK.

## Abstract

Mid-latitude Northern Hemisphere extreme cold events continue to occur despite overall winter warming trends. These events have been linked to weakened stratospheric polar vortex (SPV) states. In this study, we analyze both the upper and lower polar stratosphere for links to extreme winter cold and snow in the continental US, finding two SPV variations of interest. The first features an upper-level vortex displaced toward western Canada and linked to northwestern US severe winter weather. The second features a weakened upper-level vortex displaced toward the North Atlantic and linked to central-eastern US severe winter weather. Both variations feature lower-level stretched vortices and stratospheric wave reflection. Since 2015, a northwestward shift in severe winter weather across the US is concurrent with an increase in the frequency of the westward-focused variation relative to the eastward-focused variation and a shift to more negative phases of the El Niño–Southern Oscillation.

## Teaser

Extreme US cold linked to two variations of a stretched stratospheric polar vortex can explain a Northwest US cooling trend.

## INTRODUCTION

Climate change is projected to increase the frequency and severity of extreme weather throughout the globe, particularly in terms of heat and precipitation, based on increasing near-surface temperature and the accompanying increase in water vapor (*1*). The near-surface temperature warming is not occurring at the same rate everywhere. Warming is occurring at a substantially higher rate relative to the global average in the wintertime polar regions, in a phenomenon known as Arctic amplification (*2*). At the same time, despite the overall warming climate in the continental mid-latitudes of the Northern Hemisphere (NH), episodes of extreme winter cold and snow continue to occur in many of these mid-latitude regions (*3*, *4*). Specifically, while North America (NA) winter cold extremes have warmed faster than mean temperatures since 1980 (*5*), extreme winter cold events in the central-eastern US (CEUS) have not similarly decreased in frequency or intensity (*6*). Recent US cold extremes occurred in the winters of 2009/2010 (*7*), 2013/2014 (*8*), 2014/2015 (*9*), 2020/2021 (*10*), and 2024/2025 (*11*). The specific mechanisms for mid-latitude cold extremes may vary, but, here, we examine severe winter weather in the continental US (CONUS) in terms of its relationship to polar stratospheric variability.

Links between the stratosphere and mid-latitude weather have been recognized since the mid– to late 20th century (*12*). Sudden stratospheric warmings (SSWs) occur when the stratospheric polar vortex (SPV) absorbs upward-propagating wave energy, weakening and reversing the westerly flow. This results in a rapid warming of the polar stratosphere, which projects strongly onto the negative phase of the stratospheric Arctic Oscillation (AO) (*13*), as well as onto the negative phase of the Northern Annular Mode (NAM) (*12*, *14*) in the stratosphere. During SSWs, the weakened SPV is displaced from the Central Arctic or split into two vortices across the Eastern Hemisphere and Western Hemisphere, which can result in a downward propagation of anomalous easterlies into the troposphere and severe winter cold in the mid-latitudes, especially for Eurasia and NA (*15*–*17*). Not all SPV disruptions result in SSWs, and some NA cold-air outbreaks (CAOs) occur in the absence of SSWs [e.g., (*18*)], as happened in 2013/2014 (*5*). In those cases, extreme cold was preceded by a stretching of the SPV (where the vortex elongates from eastern Asia toward eastern Canada or US) rather than a displacement or split associated with SSWs. Stretching of the SPV is associated with a reflection rather than absorption of upward-propagating planetary waves from the stratosphere back to the troposphere. Reflection of planetary waves occurs when there is a negative vertical wind shear in the mid- to upper-level stratosphere (zonal mean winds weakening with height) coupled with a lower- to mid-level stratosphere waveguide (meridional winds weakening with latitude) (*10*, *19*–*22*). Reflection is also linked to strong static stability in the lower stratosphere (*23*, *24*).

Stratospheric reflection of planetary waves and their associated surface impacts have been examined in numerous studies (*8*, *10*, *15*–*33*). For example, Kretschmer *et al.* (*18*, *28*) investigated the role of planetary wave reflection in winter Eurasian and NA cold spells by looking at patterns of low-level stratospheric variability. They showed that NA cold-air incursions are often associated with a lower SPV pattern linked to reflected upward-propagating planetary waves originating over Siberia and downward-propagating over Canada, in conjunction with anomalous ridging over the North Pacific in both the troposphere and stratosphere. Building on this, Cohen *et al.* (*10*) demonstrated that CAOs in the central US, such as the 2021 Texas cold snap, can result from a pathway through the SPV, in which Barents-Kara Seas sea-ice melt and heavy Siberian snowfall favor a tropospheric planetary wave that results in ridging over the Urals coupled with a deep trough in eastern Asia, which can excite upward-propagating waves that converge in the northern North Pacific. These waves act to weaken and stretch the SPV in a manner conducive to downstream stratospheric wave reflection (*18*), which, in turn, enhances tropospheric wave amplification over NA and leads to cold Arctic-sourced surface air advected anomalously southward, deepening jet-stream troughs.

In addition, Messori *et al.* (*29*) found that persistent lower-stratosphere strong reflective events are often associated with an abrupt tropospheric shift from anomalous Pacific troughing to anomalous Alaskan ridging along with shifts from anomalously warm surface temperatures to anomalously cold surface temperatures over much of continental NA. Millin *et al.* (*30*) showed that the majority of extreme CAOs in the central US are associated with either anomalous tropospheric pan-Arctic ridging (similar to SSWs) or anomalous Alaskan ridging. Ding *et al.* (*31*) reported that NA anomalous cold can occur with both symmetric weak SPVs (similar to SSWs or negative North Atlantic Oscillations) and asymmetric weak SPVs (planetary wave 1 structures, similar to positive North Atlantic Oscillations). Specifically, stronger asymmetric wave 1s tend to result in stronger Pacific ridging and troughing over NA with a lead time for NA cold of 5 to 25 days, while weaker asymmetric wave 1s are often associated with near-simultaneous NA cold (*32*, *33*). Relatedly, Shen *et al.* (*23*) found an oscillating mode between the first two Empirical Orthogonal Functions (phase-shifted wave 1s) of 10- to 60-day bandpass-filtered 10-hPa geopotential heights that explain more than 50% of the intraseasonal variation, linking Rossby wave propagation between the stratosphere/troposphere and a westward-shifting SPV that facilitates vertical wave propagation that can lead to CAOs in the mid-latitudes.

Each of these examples of previous research has investigated the stratospheric link to NA CAOs from differing perspectives (CAOs, stratospheric reflection of planetary waves, and tropospheric/stratospheric Rossby wave interactions) and definitions of extremeness, with the results being largely complementary in describing several key dynamical processes and tropospheric/stratospheric interactions that precede the CAOs, including detection of wave reflection and stretched or asymmetric weak SPVs. On the one hand, this speaks to growing evidence that the stratosphere plays an important role in winter surface temperatures beyond SSWs. On the other hand, several key questions remain, including (i) how frequently does the stratosphere play a role in more frequent, shorter-duration reflective events, (ii) how important is the stratospheric influence for regions other than the CEUS, (iii) how many distinct modes of reflection exist both spatially and at different levels of the stratosphere and how do they relate to each other, and (iv) how do the events and the surface-stratosphere link change in a warming climate?

Here, we examine daily stratospheric variability at multiple vertical levels and investigate the associated impacts on US severe winter weather, the role of reflection and the relationship between different stretched vortex patterns, and the links to a range of teleconnections to address some of these outstanding questions. This study builds on previous research to contribute to our base of knowledge in three key areas. First, we consider multiple levels of the stratosphere concurrently to identify SPV variations. This is important because wave reflection requires a reflecting surface in the mid- to upper stratosphere accompanied by a waveguide in the lower to mid-stratosphere (*19*–*22*) and the surface impacts could differ based on the specific three-dimensional shape of the SPV (*23*). Second, we expand the examination of CAOs in the CEUS to also consider winter surface impacts in the western US. Third, we extend our consideration of winter extremes to include snow as well as cold air, using an index of winter weather severity for the US.

## RESULTS

### Identification of stratospheric variations

**Fig. 1.**
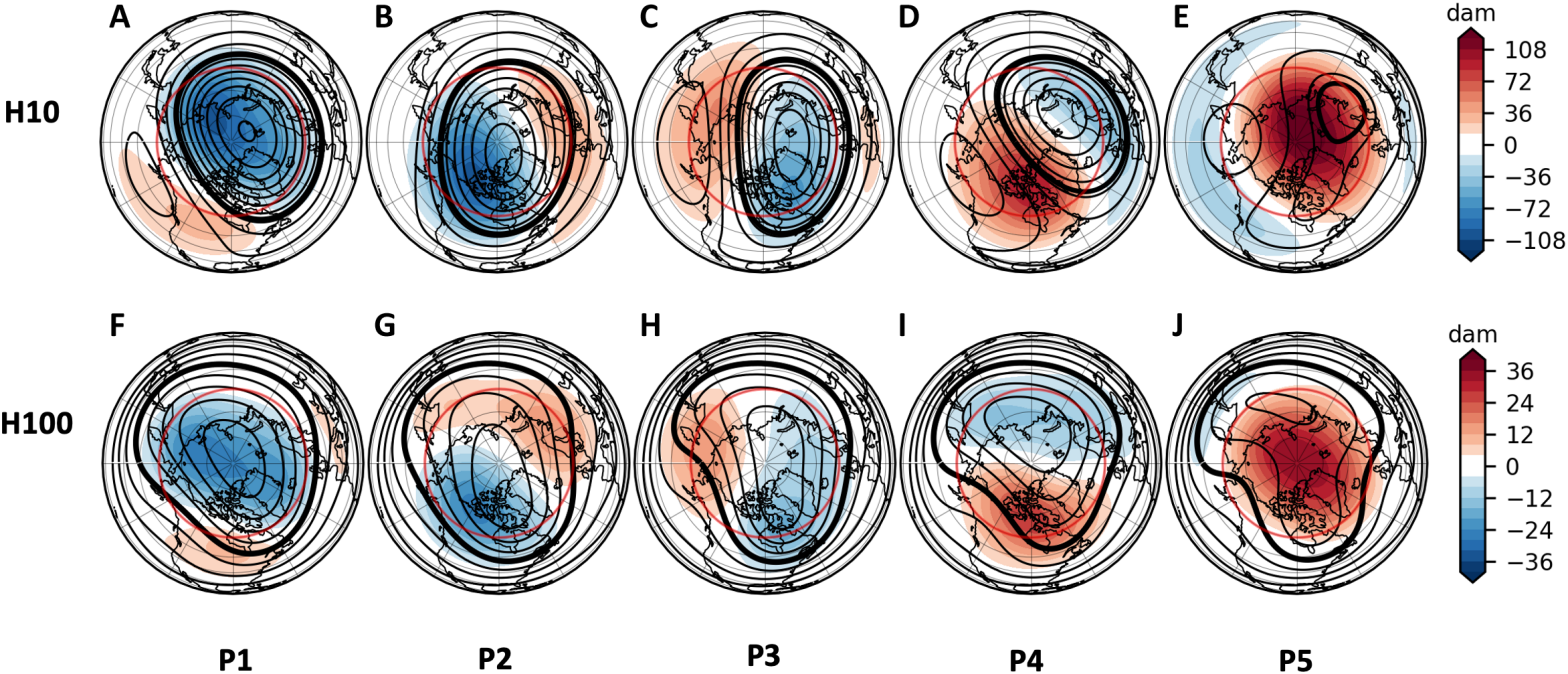
K-means results. Results of K-means clustering (*K* = 5) on 10-hPa (H10) and 100-hPa (H100) MERRA-2 geopotential height anomalies over the NH polar cap (northward of 60°N) for all January to February days from 1980 to 2021, with cluster composites shown for (**A** to **E**) 10-hPa geopotential heights (contours in 3-dam intervals; thick contour for 300 dam) and anomalies (dam, shading) and (**F** to **J**) 100-hPa geopotential heights (contours in 2-dam intervals; thick contour for 158 dam) and anomalies (dam, shading), from 20° to 90°N. The clusters are arranged from strongest 10-hPa SPV to weakest (60° to 90°N), labeled P1 to P5, respectively. The region used for clustering is indicated with a solid red line.

Separating the winter stratosphere into groupings based on upper- and lower-level characteristics can be helpful in linking stratospheric variations to specific surface conditions. To create these groupings, we perform K-means clustering of January and February 10- and 100-hPa geopotential height anomalies over the NH polar cap (see Materials and Methods), with the results shown in Fig. 1. In contrast to previous studies that have examined only one level in isolation [e.g., (*10*, *16*, *22*, *28*)], the joint clustering captures simultaneous variations in both the mid- to upper-level and lower-level stratosphere, which can help distinguish between reflecting and nonreflecting surfaces. Composites of the clusters (averages over dates with similar stratospheric patterns) are labeled left-to-right P1 to P5 and comprise 20.4, 20.1, 19.7, 20.1, and 19.7% of the total days, respectively. Here, we use the following definitions: “Cluster days” (e.g., P1 days) refers to all days assigned to a specific cluster. A “cluster event” (e.g., P2 event) refers to one or more consecutive days in the same cluster, and “cluster event days” (e.g., P2 event days) refers to all days within a specific cluster event. Occasionally, we consider only events with a duration of 3 or more days; these instances are clearly distinguished in both the text and figure captions.

The clusters display a range of upper- and lower-level vortex shapes and strength and vary from strong upper- and lower-level coupled polar vortices in P1 (mean zonal u-wind at 10 hPa and 65°N greater than 45 ms^−1^), to stretched upper and lower vortices in P2 and P3, and to weakened displaced upper and lower vortices in P4 and P5 (mean zonal u-wind at 10 hPa and 65°N less than 2 ms^−1^). In P1, the upper and lower stratosphere are tightly coupled, with the upper vortex (Fig. 1A) displaced slightly toward the Barents-Kara Seas and the lower vortex (Fig. 1F) centered over the pole. The two stretched vortices P2 and P3 have key differences at upper and lower levels of the stratosphere and between each other. In P2, the upper vortex (Fig. 1B) is centered over the pole and stronger, while in P3, the upper vortex (Fig. 1C) is displaced toward Greenland and the North Atlantic. While both P2 and P3 lower vortices are stretched from Asia to NA, the P2 lower vortex (Fig. 1G) features troughing over central NA and above-normal geopotential heights across northern Eurasia, while the P3 lower vortex (Fig. 1H) is slightly displaced toward Greenland and features strong ridging from eastern Asia to Alaska and troughing over eastern NA. The weakened upper vortices in P4 (Fig. 1D) and P5 (Fig. 1E) are displaced over eastern Europe and western Asia, while the lower vortices (Fig. 1, I and J) are both located over north-central Asia. Lower stratosphere positive height anomalies in P4 and P5 extend from the pole to eastern Canada.

Several of these clusters relate to previous research and have connections to extreme weather. The P3 lower vortex shares some similarities to a stretched low-level SPV discussed in Cohen *et al.* (*10*), which was associated with reflection and extreme CAOs in the CEUS. The P3 cluster days also considerably overlap long-duration January-to-February reflective events identified in Messori *et al.* (*29*), with more than 70% of the P3 days occurring during long-duration events. In contrast, only 20% of the P2 cluster days occur during those same longer-duration reflective events. The P4 and P5 stratospheric clusters share many similarities to stratospheric signatures of SSWs (*13*), with the P5 variation suggestive of a split SPV with a second vortex centered near Baffin Bay. Of the start dates for 18 recent SSWs from 1980 to 2021 (*34*), 8 fall into the P4 cluster and 9 fall into the P5 cluster. Moreover, most of these SSW start dates with split or near-split polar vortices occur during days assigned to the P5 cluster (five of seven).

**Fig. 2.**
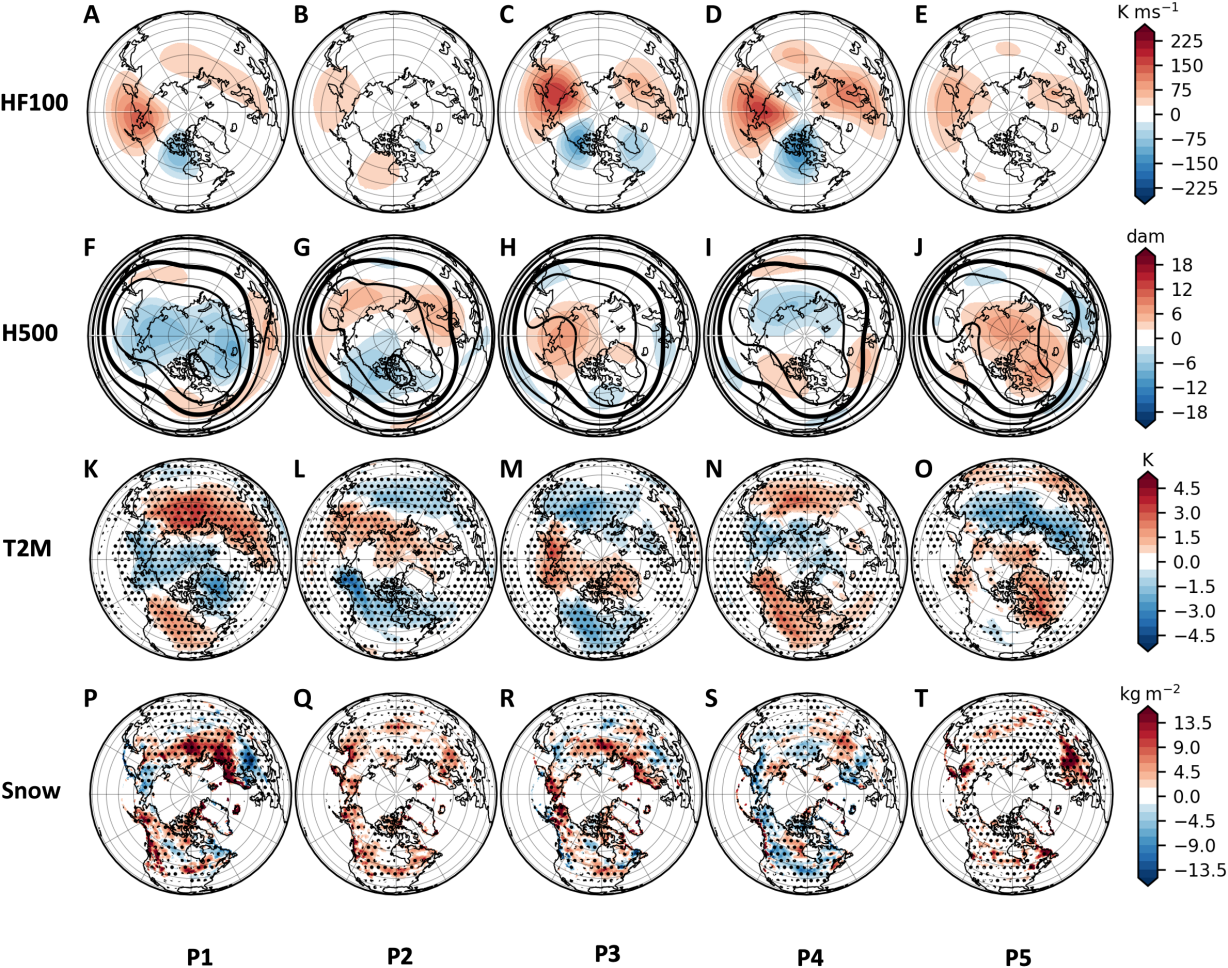
Additional cluster composites. Composites of additional MERRA-2 fields associated with the P1 to P5 cluster days from Fig. 1, including (**A** to **E**) 100-hPa heat flux (HF100; K ms^−1^, shaded), (**F** to **J**) 500-hPa geopotential heights (H500; dam, contours every 20 dam with thick contour for 540 dam, and anomalies shaded), (**K** to **O**) 2-m temperature (T2M) anomalies (K, shaded), and (**P** to **T**) surface snow mass anomalies (kg m^−2^, shaded). In (K) to (T), stippling indicates grid values significant at the 0.05 level based on bootstrapping.

These five patterns (based on joint clustering of two stratospheric levels) can give insights into related tropospheric and surface conditions. In Fig. 2, additional composites of the lower stratosphere and troposphere are shown for the cluster days. First, we consider lower stratospheric poleward heat flux (100 hPa V′T′ where the primes indicate departures from the daily zonal means of meridional wind and temperature at each grid point; Fig. 2, A to E), which we use as a proxy for upward wave flux activity (*28*) to identify heat flux dipoles. Heat flux dipoles, with positive heat flux in one region and negative heat flux in an adjacent or nearby region, can indicate stratospheric reflection, whereby upward wave activity, instead of passing from the troposphere into the upper stratosphere, can be deflected downward in the stratosphere toward the troposphere and lead to various surface impacts, including anomalous warmth or cold (*4*, *8*, *10*, *18*, *21*, *25*–*28*, *32*). Heat flux dipoles are similar for P1 (Fig. 2A) and P4 (Fig. 2D), with positive heat flux (upward wave activity) over Alaska and the North Pacific and negative heat flux (downward wave activity) over central Canada. P2 (Fig. 2B) and P5 (Fig. 2E) have positive heat flux centered over eastern Asia and, in the case of P2, also over central Canada. P3 (Fig. 2C) has a similar heat flux dipole to P1 and P4, but the upward wave activity is shifted westward over eastern Asia, and the downward wave activity occurs over Alaska. As previously noted, the P3 cluster shares some similarities to the reflective stretched SPV patterns identified in Cohen *et al.* (*10*) and Messori *et al.* (*29*), which were associated with extreme cold in the central US. While we will, at times, compare this study’s P3 cluster to the similar cluster from Cohen *et al.* (*10*), it is important to note that the clusters are not identical, because our P3 cluster is based on clustering of both the upper and lower SPVs, while that in Cohen *et al.* (*10*) was based on the lower SPV (i.e., 100-hPa geopotential height anomalies) only.

Composited geopotential height anomalies at 500 hPa (Fig. 2, F to J) show the most negative height anomalies associated with the P1 cluster (mean anomaly of −48 m between 70° and 90°N) and the most positive height anomalies associated with the P5 cluster (mean anomaly of 56 m between 70° and 90°N). The other three clusters show trough/ridge anomalies in different mid-latitude locations: for P2, above-normal geopotential heights stretch across Europe and Asia, with troughing over western Canada; for P3, ridging is centered over the Dateline and troughing is shifted to eastern Canada; and for P4, weak ridging is located over western Canada and Greenland, with troughing over central-eastern Asia. The P3 500-hPa height field is similar to the Alaskan Ridge weather regime, which has been shown to have connections to US cold events via stratospheric wave reflection (*30*, *35*–*37*).

Composited surface conditions associated with each cluster are shown in Fig. 2 (K to O; temperature) and Fig. 2 (P to T; snow mass). The P2 and P3 clusters are related to the most anomalous winter weather (cold temperatures and snow) in the CONUS. In P2 (Fig. 2, I and Q), a swath of anomalous snow and cold extends across the north-central CONUS, with interior Asia anomalously cold and central Arctic and northeast Asia anomalously warm. In P3 (Fig. 2, M and R), CONUS cold air extends from the northwestern US (NWUS) to Florida, with anomalous snow in the central and eastern US. In contrast to P2, anomalously warm Arctic temperatures are shifted toward Alaska and Greenland, and anomalously cold Eurasia temperatures are shifted northeastward toward Siberia and eastern Asia in P3. P1 (Fig. 2K) and P4 (Fig. 2N) have anomalously warm conditions over mid-latitude Asia and NA, although P4 is also associated with cold conditions limited to Siberia, while P5 has anomalously cold conditions over northern Eurasia and warm anomalies over Greenland. As noted earlier, SSWs tend to occur during P4 and P5 stratospheric cluster days.

### Stratospheric variations and CONUS severe winter weather

**Fig. 3.**
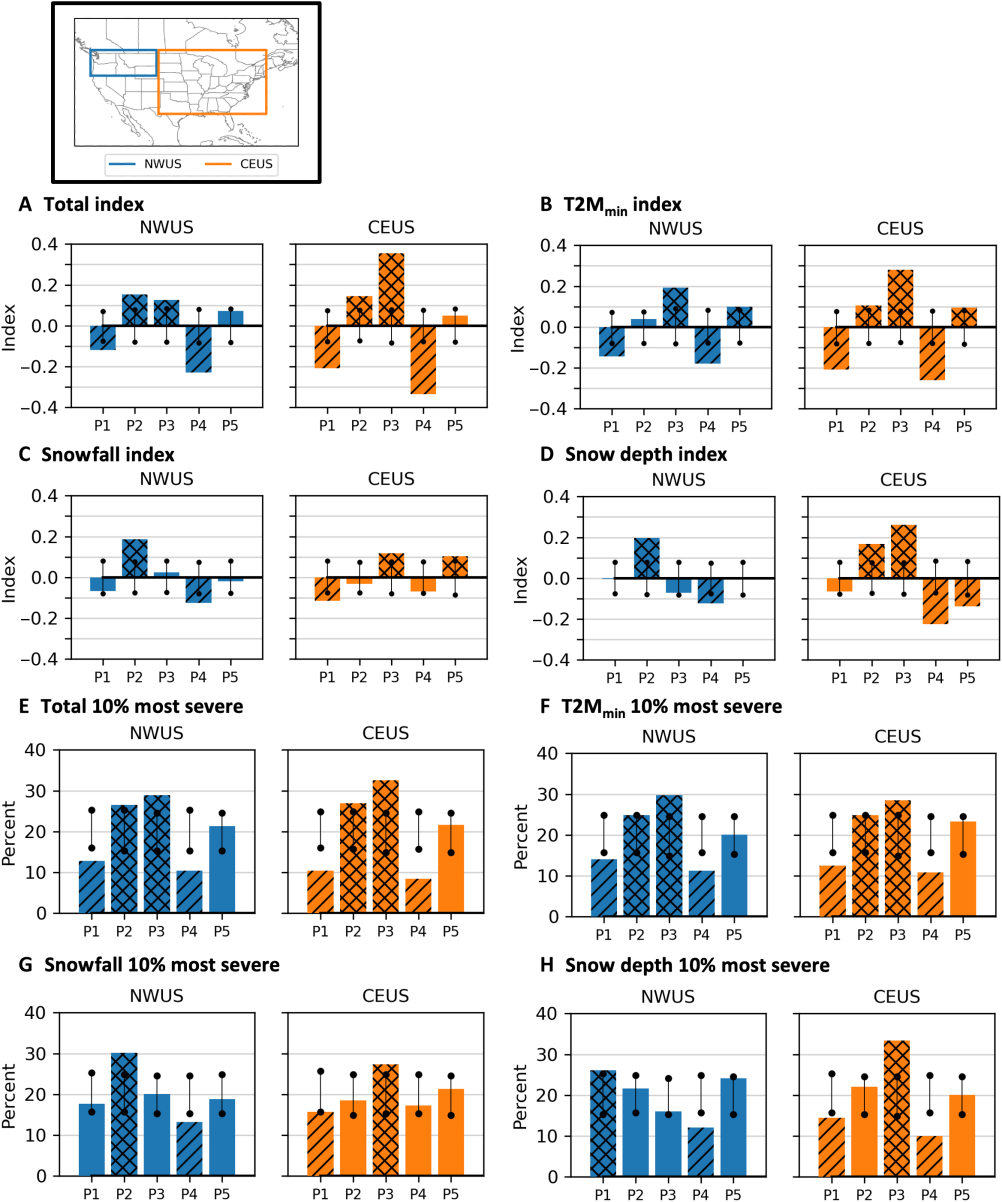
Severe winter weather index. Composites of temporal mean latitude-weighted area-averaged rAWSSI on P1 to P5 cluster days for the two selected US regions shown in the inset (NWUS and CEUS), using (**A**) the total index, (**B**) 2-m minimum temperature index values, (**C**) snowfall index values, and (**D**) snow depth index values. Bottom panels show the percentage of P1 to P5 days, respectively, assigned to the most extreme 10% of daily (**E**) total index values, (**F**) 2-m minimum temperature index values, (**G**) snowfall index values, and (**H**) snow depth index values. Black vertical lines indicate the 95% confidence interval of the index value if the values were randomly sampled from all days (bootstrapping 1000 times). Composite index values (bars) that fall outside the vertical lines are double hatched if larger (more severe or larger) than the expected range and single hatched if smaller (less severe or smaller) than the expected range based on bootstrapping.

There are two clusters or regions of interest in the CONUS where anomalous cold and snow are primarily located: the NWUS for the P2 cluster and the CEUS for the P3 cluster (the NWUS and CEUS boundaries are shown in Fig. 3, inset). Thus, the remainder of this paper will concentrate on examining the stratospheric variations in P2 and P3 and their related surface impacts in these two regions. Surface impacts in these regions are examined using the reanalysis-based Accumulated Winter Season Severity Index (rAWSSI; see Materials and Methods) as defined in Mayes Boustead *et al.* (*38*) and used in Cohen *et al.* (*6*). The rAWSSI is a gridded metric for winter severity that takes into account accumulated temperature, snowfall, and snow depth, both separately and combined. The NWUS and CEUS composite index values for each set of cluster days are shown in Fig. 3 (A to D). The combined index shows an increased likelihood of severe winter weather for both the P2 and P3 stratospheric variations in both regions (Fig. 3A). For the NWUS, there is a statistically significant increase in severe cold during P3 days (left panel of Fig. 3B) and severe snow during P2 days (left panels of Fig. 3, C and D). For the CEUS, the primary cluster associated with both rAWSSI severe cold (right panel of Fig. 3B) and snow (right panels of Fig. 3, C and D) is P3, although the P2 cluster is also strongly associated with above-normal snow depth.

In addition, we examine which stratospheric variations are associated with the most severe winter weather in each region (Fig. 3, E to H). Using the surface temperature index, the most severe (highest) 10% of regional daily rAWSSI values tend to fall into P2 and P3 for the NWUS and P3 for the CEUS. The most severe snowfall occurs in P2 for the NWUS and P3 for the CEUS. Extreme snow depth is associated with P3 in the CEUS. Regardless of the approach, whether by examining composited anomalies of reanalysis surface temperature and snow mass (as in Fig. 2, K to T) or using standardized severity measures such as the rAWSSI, both the P2 and P3 stratospheric variations show a strong association with severe winter weather in the CONUS.

While our focus is on severe winter weather associated with the P2 and P3 clusters, we also note that clusters P1 and P4 stand out for having the least overall severe winter weather in both regions (Fig. 3, A and E). The P5 cluster is noteworthy for its relationship to severe cold in both regions of the CONUS as well (Fig. 3B), likely related to the downward propagation or influence of the negative phase of NAM into the mid- to lower troposphere during SSWs, but this relationship is less robust than for other areas of the NH such as northern Eurasia (*15*, *16*, *18*, *22*).

### Wave reflection in stratospheric variations

Both the reanalysis temperature and snow mass composites (Fig. 2, K to T) and the rAWSSI indices (Fig. 3) confirm that the days assigned to clusters P2 and P3 are associated with CAOs and/or snow events for the CONUS, with P2 days related mainly to NWUS severe winter weather and P3 days related mainly to CEUS severe winter weather. The stratospheric/tropospheric interactions involved with the regional severe weather outbreaks are likely quite different, as seen by the differences in composites of P2 and P3 clusters at various atmospheric levels in Figs. 1 and 2. As noted earlier, the P3 lower stratosphere shares similarities to that explored in Cohen *et al.* (*10*). In that study, a stretched lower SPV was associated with the reflection of wave activity flux (WAF), which coincided with anomalous ridging over eastern Asia and the North Pacific and troughing over NA. Here, P3 (Fig. 2C) has a 100-hPa positive heat flux (upward wave activity) centered over eastern Asia and negative heat flux (downward wave activity) centered over northwestern Canada. This positioning of the heat flux dipole coincides with strong ridging in the North Pacific and a downstream trough in the lower stratosphere and troposphere and thus may be linked to the subsequent strong incursion of cold air into the CEUS. Conversely, in P2 (Fig. 2B), 100-hPa areas of positive heat flux over both the North Pacific and Canada are not consistent with concurrent wave reflection.

**Fig. 4.**
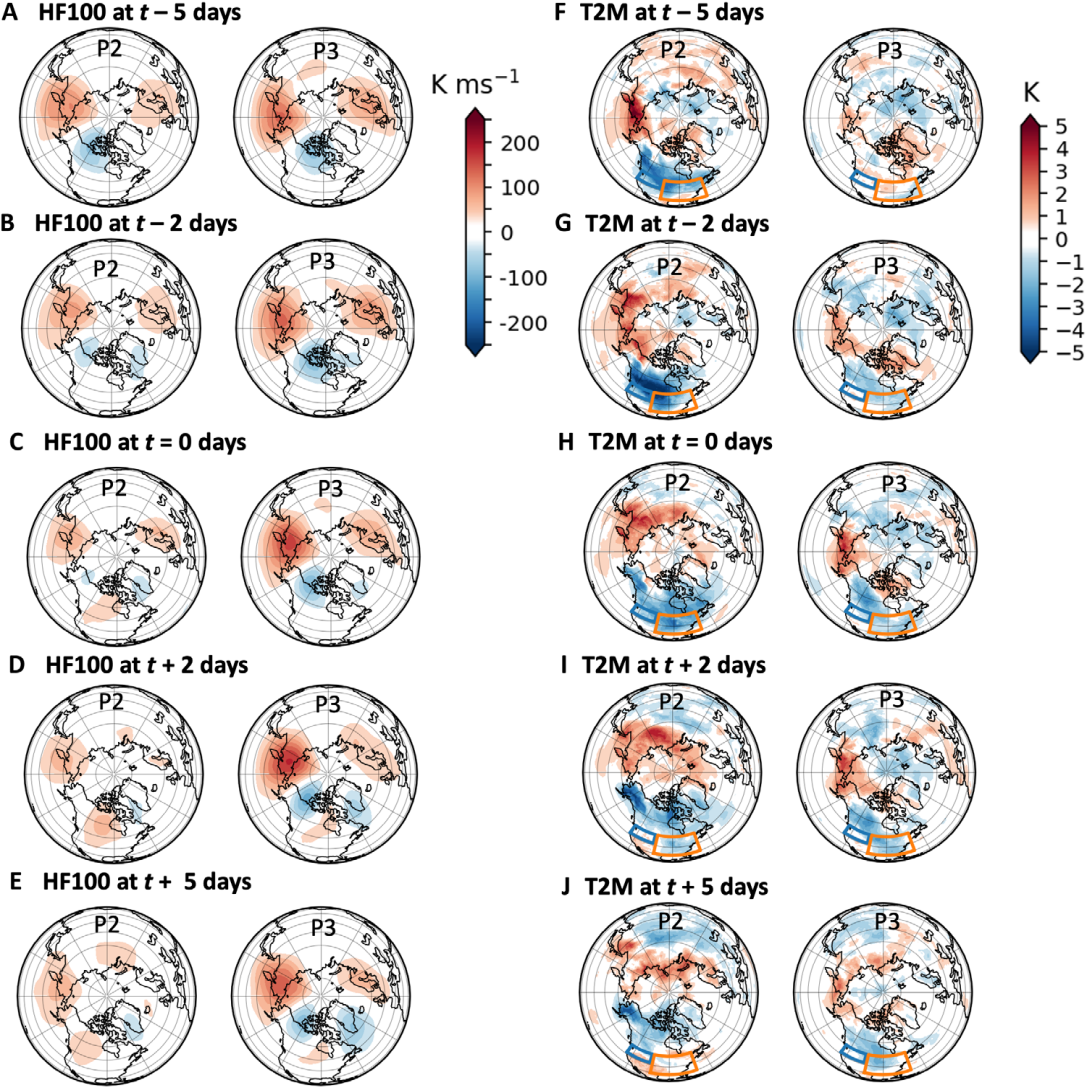
Reflective dipole associated with cluster events. MERRA-2 100-hPa heat flux (HF100; K ms^−1^) for P2 and P3 events at various leads/lags of event onsets: (**A**) *t* – 5 days, (**B**) *t* – 2 days, (**C**) *t* = 0 days, (**D**) *t* + 2 days, and (**E**) *t* + 5 days. (**F** to **J**) Similarly, MERRA-2 2-m surface temperature anomalies (T2M; K) for P2 and P3 events at the same leads/lags and showing the outline of the NWUS (blue) and CEUS (orange) regions from Fig. 3. P2 and P3 events are defined as three or more consecutive days within each cluster.

To explore this further, we show composites of heat flux for the start of P2 and P3 events of at least 3-day duration adjusted for various lead/lag times (Fig. 4, A to E). For P2 events, a weak heat flux dipole is apparent 5 days before the event start (Fig. 4A), indicated by positive heat flux over Siberia and eastern Asia and negative heat flux over Alaska. This dipole disappears immediately preceding and after the start of P2 events (Fig. 4, B to E). This suggests that reflection of upward wave activity may play a role in the onset of P2 events, rather than during P2 events. For P3 events, a strong heat flux dipole exists for eastern Asia into western NA both preceding and following (Fig. 4, D and E) the start of the events; however, the location of the dipole moves westward and strengthens during this time. Specifically, at 5 days before the start of P3 events (Fig. 4A), positive heat flux over the North Pacific is coupled with negative heat flux over central Canada. By the start of P3 events (Fig. 4C), the center of the positive heat flux occurs over eastern Asia and the center of the negative heat flux occurs over Alaska. Following the start of P3 events (Fig. 4E), the dipole remains in this position and strengthens further.

The corresponding surface temperatures are shown in Fig. 4 (F to J) for various lead/lag times surrounding the start of P2/P3 events of at least 3-day duration. For P2 events, NWUS and CEUS surface air is anomalously cold at least 5 days before the start of the event (Fig. 4F), and this cold air moderates somewhat within a few days after the start of the event (Fig. 4, I and J). For P3 events, anomalously cold air begins to appear 2 days before the start of the event (Fig. 4G) in the eastern US, transitions to the CEUS and NWUS by the start of the event, and then remains in place following the start of the event (Fig. 4, I and J). For the P3 events, the heat flux dipole and corresponding surface temperatures at various lags are consistent with the long-duration reflective events examined in Messori *et al.* (*29*).

**Fig. 5.**
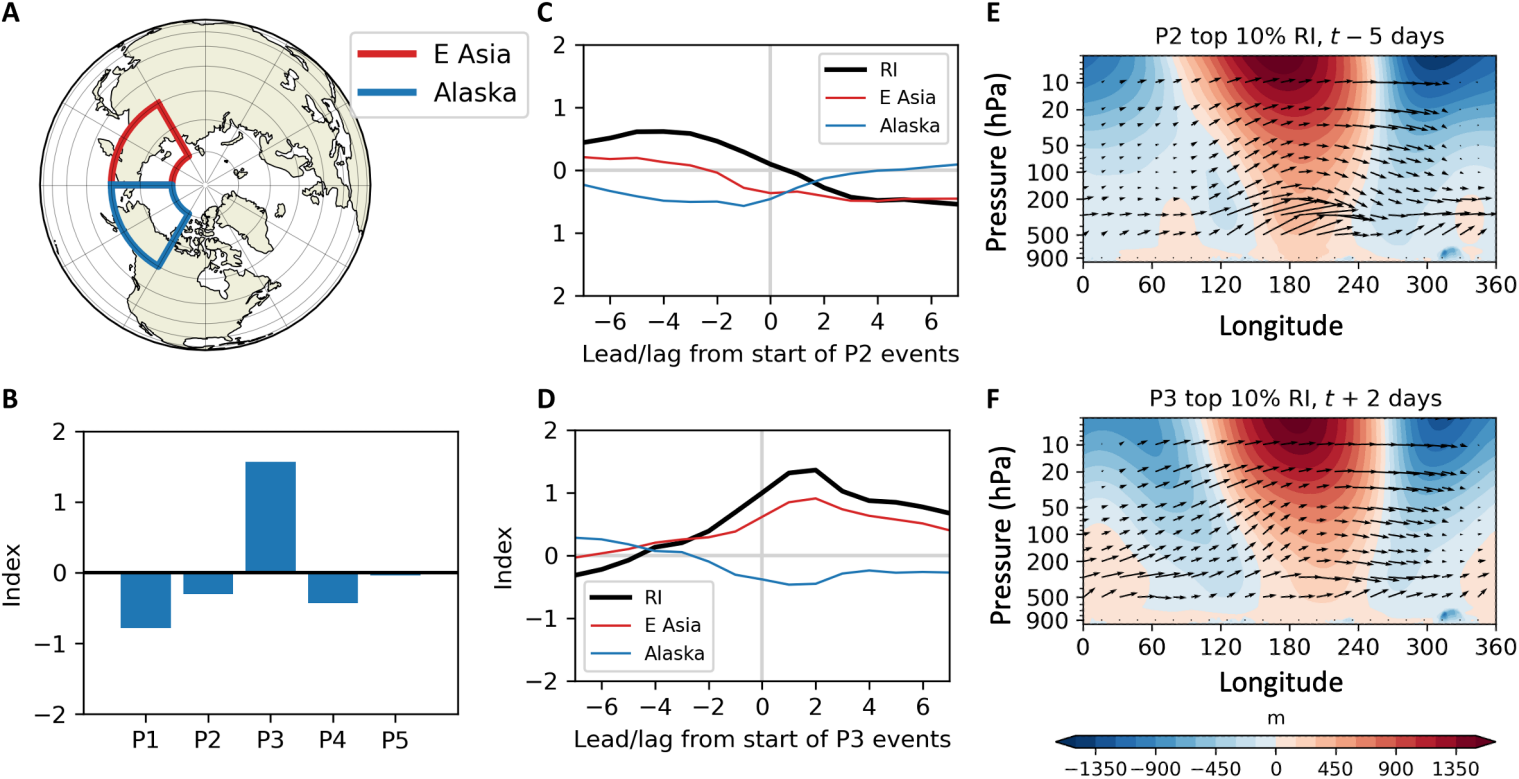
Simple reflective index. (**A**) Regions for analyzing reflective dipole and (**B**) simple reflective index composited by cluster days, where the reflective index is the latitude-weighted area-averaged 100-hPa MERRA-2 poleward heat flux over eastern Asia (E Asia) minus that over Alaska, standardized for all days of January to February 1980 to 2021. Positive values of the index indicate upward wave activity over eastern Asia and downward wave activity over Alaska. (**C** and **D**) The simple reflective index (RI) at various leads/lags is composited from the onset of P2 and P3 events, respectively. The eastern Asian (red line) and Alaskan (blue line) components of RI are also shown. (**E** and **F**) Cross sections of area-averaged 55° to 75°N eddy geopotential heights (m, shading) and WAF (vectors, filtered for the first three wave numbers) are shown for the top 10% daily reflective indices of P2 event onsets, 4 days before onset to match the optimum window for reflection, and P3 event onsets, 2 days after onset to match the optimum window for reflection, respectively. P2 and P3 events are defined as three or more consecutive days within each cluster.

We further explore the role that reflection may play in the subsequent surface conditions for P2 and P3 events by creating a simple reflective index, similar to that used in previous studies (*22*, *29*, *30*), but for the regions identified in Fig. 5A. The reflective index is defined as the daily difference between the 100-hPa latitude-weighted area-average standardized heat flux anomalies over northeastern Asia (red outline) and Alaska/western Canada (blue outline). Large positive values of the index may indicate upward wave activity over Asia and downward wave activity over Canada. The mean reflective index composited for the days within each cluster event is shown in Fig. 5B, with strong reflection associated with P3 events only. Figure 5 (C and D) shows the mean index value for the days surrounding the start of P2 and P3 events, confirming that reflection of stratospheric waves regularly occurs before the start of P2 events and shortly after the beginning of P3 events. The reflection after the onset of a P3 event is stronger than that preceding the onset of a P2 event. In Fig. 5 (E and F), we confirm the presence of reflection by examining a composite of 55° to 75°N cross sections of eddy geopotential heights and total-field WAF for the top 10% of daily reflective indices at the start of each cluster event, at a lead time of 5 days for P2 events and a lag time of 2 days for P3 events, consistent with Fig. 5 (C and D). Upward wave activity is present between 120° and 180°E in conjunction with a westward tilt with the height of the geopotential anomalies and between 180° and 250°E in the presence of an eastward tilt with height. The reflective surface for P2 event start days is strongest in the lower stratosphere, while that for P3 event start days extends into the middle stratosphere.

Figure 5 suggests that reflection of WAF can play a role in severe winter weather related to both P2 and P3. For P2 events, wave reflection before the event (Fig. 4A) sets up anomalously cold air at the surface in the NWUS several days before the event (Fig. 4F), and, although this cold surface air moderates toward the start of P2 events, it can result in anomalous snowfall in the NWUS during P2 events (Fig. 3, C and D). For P3, wave reflection during the event (Fig. 4, C and D) results in anomalously cold temperatures and snowfall for the CEUS (Fig. 3, B to D).

**Fig. 6.**
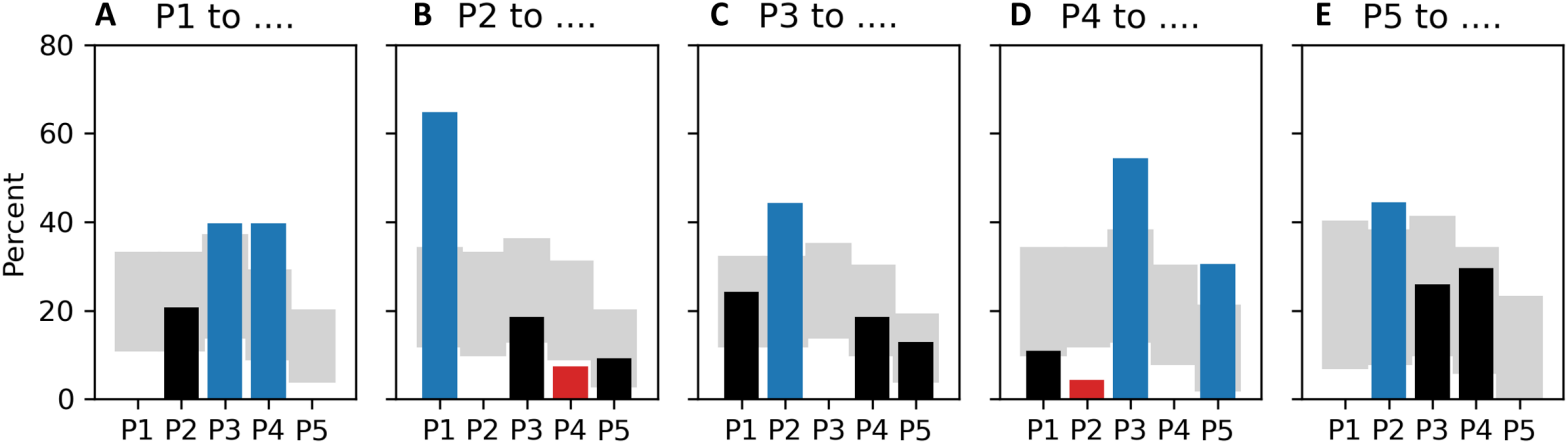
Transitioning of cluster events. Percentage of events (January to February 1980 to 2021) that transition to each of the other clusters for (**A**) P1, (**B**) P2, (**C**) P3, (**D**) P4, and (**E**) P5 events. For example, in (A), P1 events transition to P2 events 20% of the time, to P3 events 40% of the time, etc. The gray background indicates the 95% confidence interval of the rate of transitioning to any other cluster purely due to chance, on the basis of random sampling of all events (bootstrapping 1000 times). Bars are blue if the percentage is higher than expected due to chance, red if lower than expected due to chance, and black otherwise.

Because P3 events feature strong stratospheric reflection and P2 events are often preceded by stratospheric reflection, we are motivated to ask how often P3 events precede P2 events. In this case, we consider events of any duration (we include single- and 2-day events). Figure 6 shows the frequency that cluster events transition to in each of the other clusters. For example, in Fig. 6A, P1 events are more likely than expected due to chance to transition to P3 (40%) and P4 (40%) events. From Fig. 6C, P3 events are more likely than expected due to chance to transition to P2 events (44%). This underscores the importance that stratospheric reflection can play in the role of North American cold extremes, either directly (in P3 events) or indirectly by priming the atmosphere and surface for extreme winter weather (in P2 events).

From Fig. 6, it is also apparent that other transitions from one type of stratospheric variation to another are favored. If we consider a strong polar vortex, such as P1, as a starting condition, we see that P1 events often transition to either P3 (reflective, cold CEUS) or P4 (weak SSWs) events. P4 events (most common to Canadian warmings; Fig. 2N) often transition back to P3 events or to P5 events (stronger SSWs). Both the P3 (reflective) events and P5 (SSW) events often transition to P2 (NWUS cold) events, which then transition back to P1 events. Hence, we can consider two recurrent pathways connecting the stratospheric variations: The first pathway P1➔P4➔P5➔P2 (or the related P1➔P4➔P3➔P2) is linked to weakened SPVs (including SSWs) and anomalously warm surface temperatures over NA before transitioning to colder CEUS/NWUS temperatures, while the second pathway P1➔P3➔P2 directly leads to anomalously cold surface temperatures over NA, starting in the CEUS and ending in the NWUS as well. Transitional pathways have been considered by others: Millin *et al.* (*30*) noted a tendency for their Arctic High regime (similar to P4 and P5 days) to precede their CAOs in the central US, while Shen *et al.* (*23*) described an upper-level stratospheric oscillating mode that facilitates upward wave propagation and subsequent mid-latitude CAOs, both of which are consistent with the results here.

### Temperature trends and the role of teleconnections

**Fig. 7.**
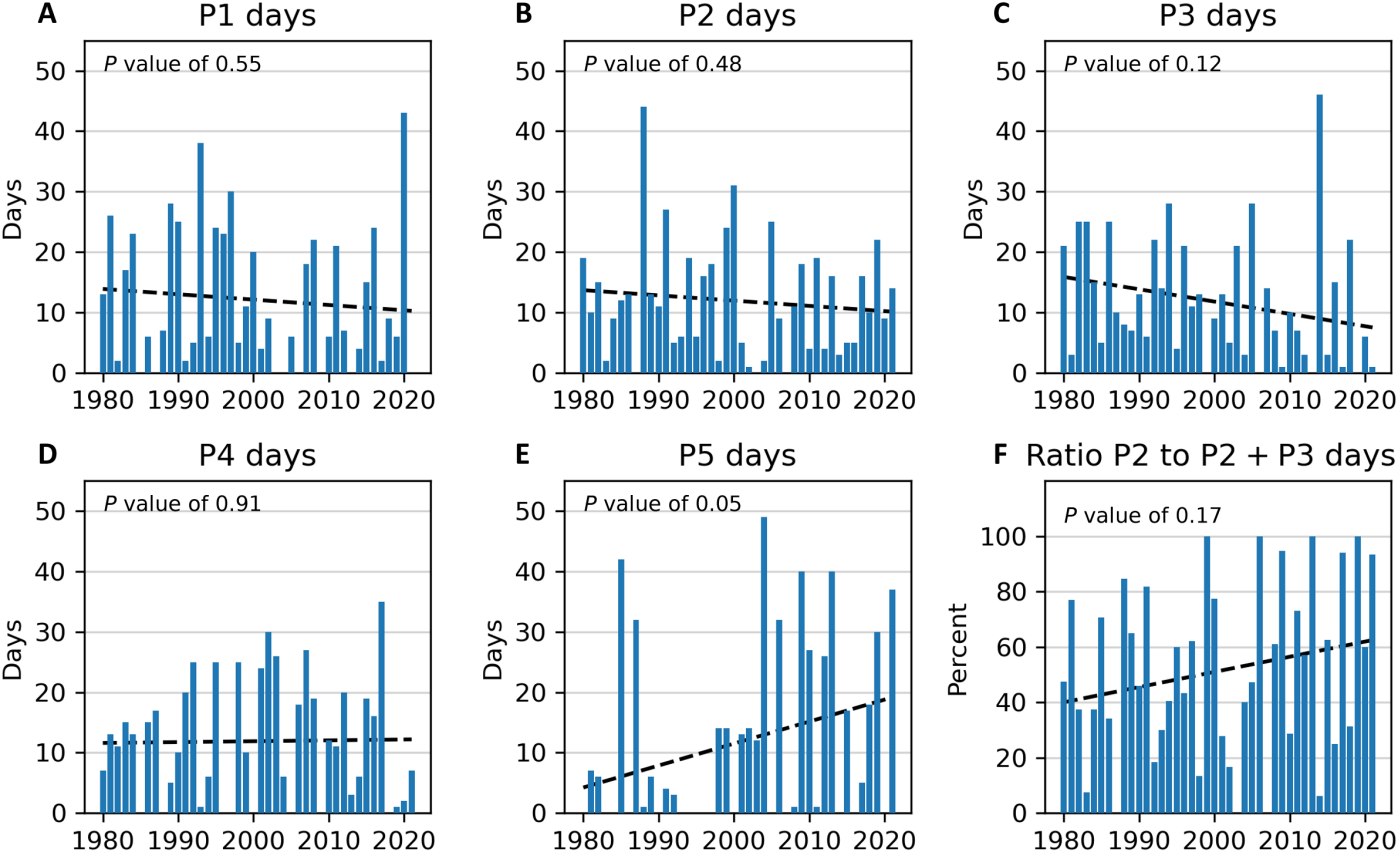
Trends in yearly cluster days. Number of January to February cluster days from 1980 to 2021 (blue bars) with trend (dashed line) for (**A**) P1, (**B**) P2, (**C**) P3, (**D**) P4, and (**E**) P5 clusters. (**F**) Ratio of January to February P2 days to combined P2 and P3 days (blue bars) with trend (dashed line). For each panel, the significance (*P* value) of the trend (see Materials and Methods) is noted at the top.

Figure 7 shows yearly trends in the number of days assigned to each cluster. No trend is discernable for the P1 (Fig. 7A), P2 (Fig. 7B), and P4 (Fig. 7D) frequency of cluster days; however, there is a 3-day-per-decade increase (at the 0.05 level of significance) in the frequency of P5 days (Fig. 7E) and a 2.5-day-per-decade decrease in the frequency of P3 (Fig. 7C) days (though not statistically significant). The ratio of P2 days to combined P2 and P3 days shows a modest increase of 0.5% per year (5% per decade; *P* = 0.17) (Fig. 7F). In other words, for the two stratospheric variations related to severe CONUS winter weather, there appears to be a recent shifting in the balance toward the cluster related to NWUS cold and away from the cluster associated with CEUS cold.

**Fig. 8.**
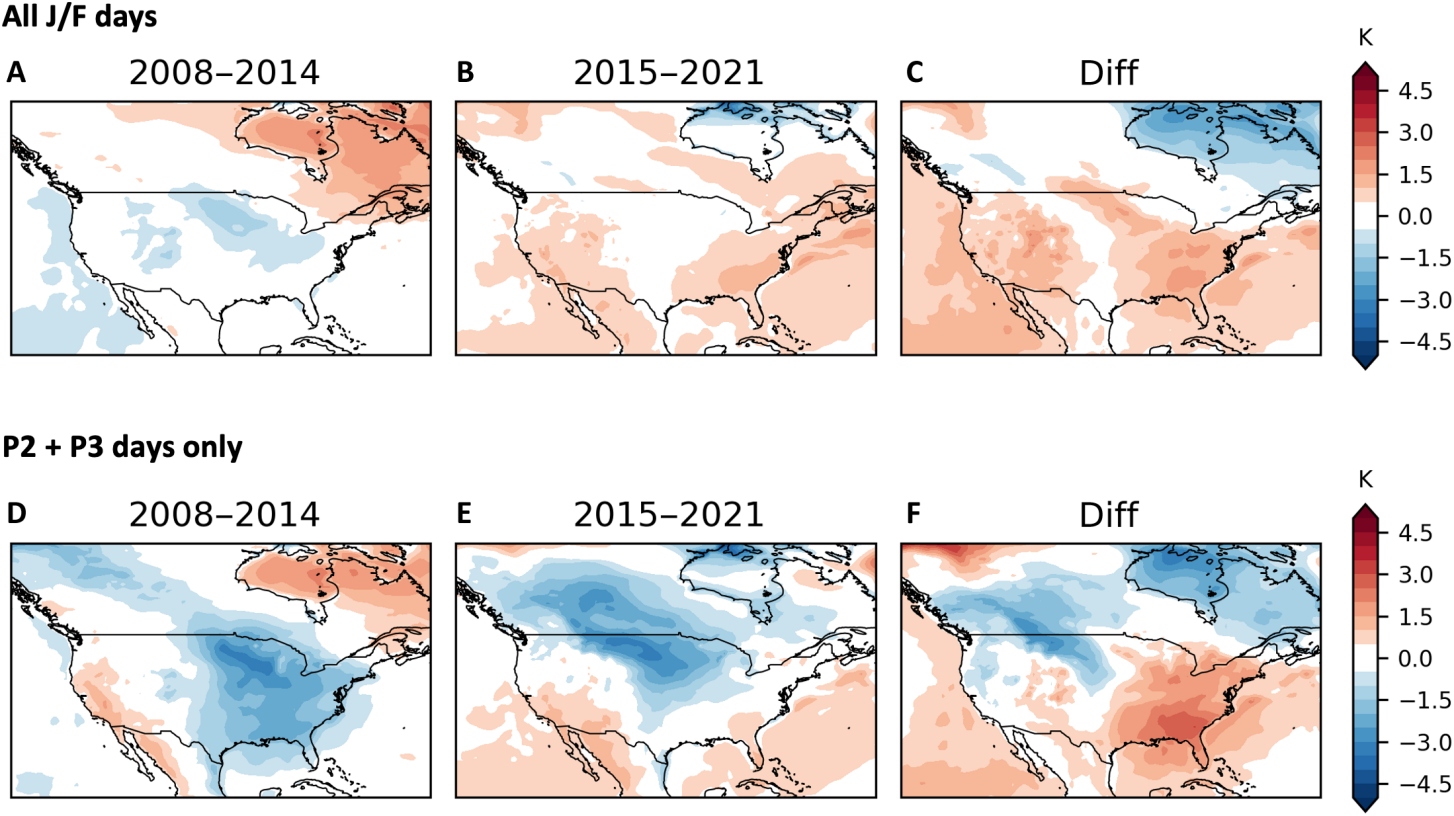
Surface temperatures across two time periods. Composite MERRA-2 2-m temperature anomalies (K) for (**A**) all days from 2008 to 2014 and (**B**) 2015 to 2021 and (**C**) the difference (latter period minus earlier period). J/F, January/February. (**D**) to (**F**) are similar to (A) to (C) except for P2 and P3 events only. P2 and P3 events are defined as three or more consecutive days within each cluster.

In Fig. 8 (A to C), we show composites of North American 2-m temperature anomalies for two periods in the recent past, January to February 2008 to 2014 and January to February 2015 to 2021, and the difference between them. There are cool anomalies in the central US for the earlier period followed by warm anomalies in the eastern and western regions of the CONUS during the later period. However, if we consider only P2 and P3 events of at least 3-day duration (Fig. 8, D to F), there is cold air in place in the eastern CONUS during the former period, but this location of cold air shifts to the central and northwest CONUS, as well as west-central Canada, during the latter period. The movement of the location of coldest air northwestward within these SPV variations coincides with the increase in ratio of P2 event days to P3 event days during the same period (Fig. 7F). In addition, during this time (since 2008) when NA has experienced a northwestward shifting in the occurrence of CONUS winter cold extremes, 64.3% of the January to February 2008 to 2021 Oceanic Niño Index (ONI) values (which measure the 3-month rolling average temperature anomalies in the east-central tropical Pacific) are negative during this period, indicative of La Niña.

Whether this recent cooling trend is related to El Niño–Southern Oscillation (ENSO) is unclear, but the role that teleconnections may play can be examined in more detail. In addition to the ONI to capture ENSO variability, we examine several other indices: (i) the Quasi-Biennial Oscillation (QBO) index, which measures the periodic (~2.5 years) shift in tropical stratospheric zonal winds between easterly (negative phase) and westerly (positive phase); (ii) the Pacific Decadal Oscillation (PDO), which captures occurrences of anomalously cool interior North Pacific sea surface temperatures (SSTs) coupled with anomalously warm Pacific coastal SSTs (positive phase) and, conversely, anomalously warm Pacific SSTs coupled with anomalously cool Pacific coastal SSTs (the negative phase); and (iii) the AO/NAM, which is based on the 1000-hPa height field between 20° and 90°N and, in its positive phase, favors a cool Arctic and relatively warm mid-latitudes, but favors a warm Arctic and relatively cool mid-latitudes in its negative phase.

**Fig. 9.**
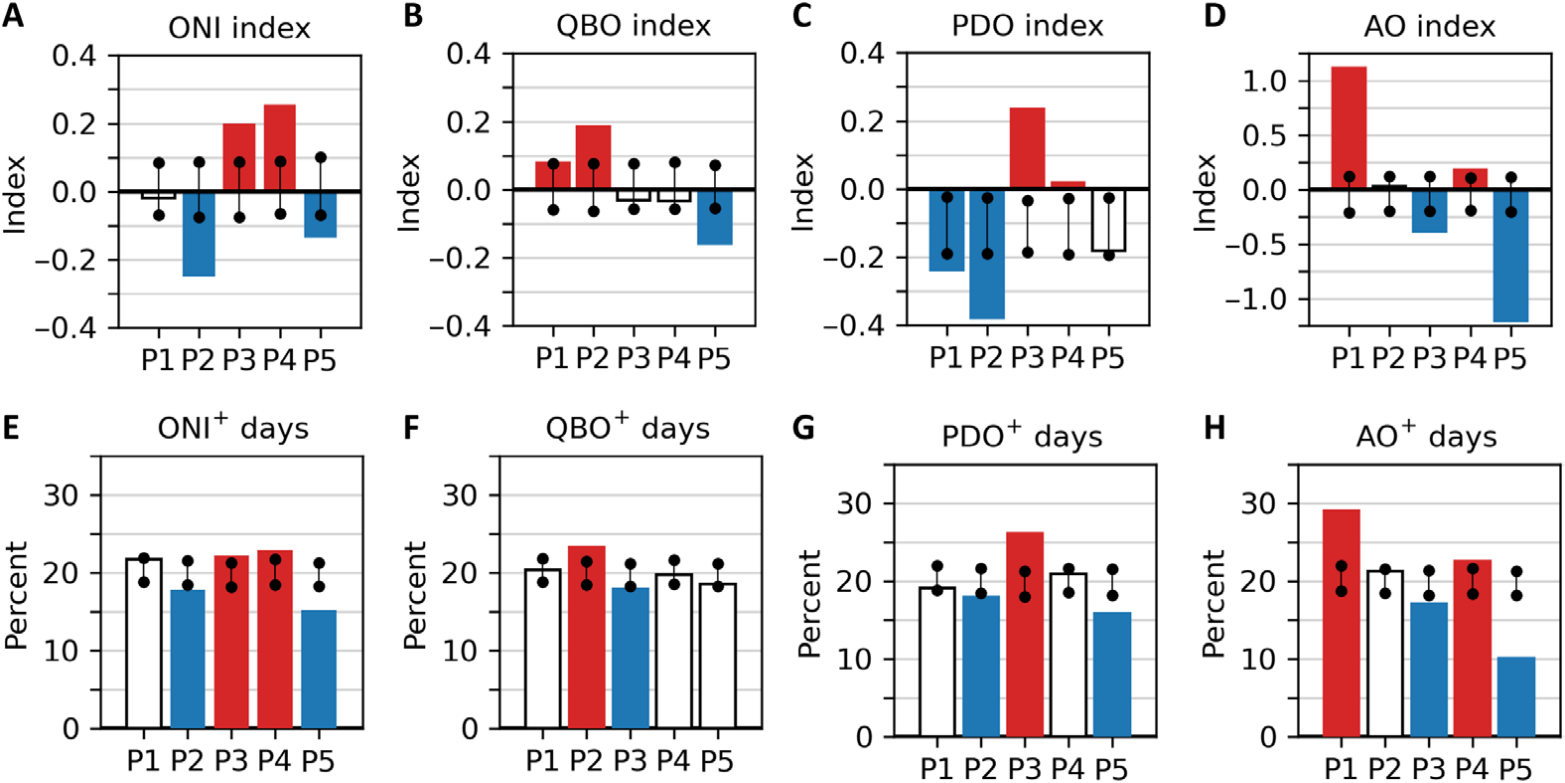
Teleconnection links to cluster days. Mean teleconnection index associated with the days in the clusters for (**A**) ONI, (**B**) QBO, (**C**) PDO, and (**D**) AO. For ONI, QBO, and PDO, the associated monthly index value is assigned to each day; for AO, the daily index value is assigned to each day. Percentage of days within each cluster when the index value is positive (greater than 0) for (**E**) ONI, (**F**) QBO, (**G**) PDO, and (**H**) AO. The vertical black lines show the 95th percentile confidence interval based on bootstrapping 1000 times. Values that fall outside that confidence interval are shown with colored bars (red if higher than the expected range, blue if lower than the expected range, and white otherwise).

In Fig. 9, we composite the four teleconnection index values associated with the cluster days. For the ONI, QBO, and PDO, the associated monthly values of the indices are assigned to each day, while for the AO, daily values are used. Once again focusing only on P2 and P3 days, the mean ONI value (Fig. 9A) for the days that make up the clusters is significantly more negative than due to chance in P2 and significantly more positive than due to chance in P3. This suggests that the strength of ENSO phases may play a role in the frequency of P2 and P3 variations and possibly the occurrence of CAOs in P2 and P3. The QBO (Fig. 9B) is significantly positive (westerly) for P2, consistent with the western phase’s tendency for colder and wetter winters in the northern US. However, P3 days are not similarly associated with either the western or eastern phase of the QBO. The PDO (Fig. 9C) is significantly negative for P2, consistent with the typical PDO^−^ signature of cool SST anomalies along the western NA coastline and above-average sea level pressure in the North Pacific, and positive for P3 days, consistent with warm SST along the western NA coastline and lower-than-average sea level pressure in the North Pacific. The AO (Fig. 9D) is significantly negative during P3 days, consistent with the typical AO^−^ signature of a warmer Arctic, more southerly NH jet stream, and more frequent central and eastern US CAOs. In Fig. 9 (E to H), we examine the frequency of positive index days (index greater than 0) for each cluster. The values in Fig. 9 (E to H) are consistent with the overall index values in Fig. 9 (A to D), suggesting that the more frequent the index sign (positive or negative) is within the cluster, the more likely the index value is lower or higher than expected.

While the teleconnection sign characteristics and impacts are largely consistent with the tropospheric/stratospheric variations seen here and the potential occurrence of severe winter weather associated with P2 and P3 events, it is not clear to what extent the stratospheric cluster variations are a response to these remote forcings. While ENSO has a strong relationship to the CONUS troposphere (*39*), the relationship between ENSO and the zonal mean polar cap vortex state has been shown to be weak over the time period of this study (*40*) and might involve nonlinearities (*41*). Moreover, the relatively short time frame examined here (1980 to 2021) is not long enough to fully understand the relationship to NA mid-latitude CAOs, particularly for decadal features such as the PDO.

## DISCUSSION

Here, we show that US winter extremes of cold and snow are associated with variations in the SPV. Using K-means clustering of 100- and 10-hPa geopotential polar cap heights, we identify five two-level stratospheric variations where the upper-level vortex can be different in shape and strength than that of the lower stratosphere. For instance, the P2 variation features a strong upper vortex and stretched wave 1 lower vortex, while the P4 variation features a strong displaced upper vortex and a weak stretched lower vortex with ridging over Alaska. Two of the five variations are associated with severe winter weather in the CONUS, and both feature lower-level weakened and stretched vortices, but they also exhibit differences. P2 features an upper-level vortex displaced slightly toward western Canada and is associated with severe winter weather in the NWUS. Meanwhile, P3 features a weakened upper-level vortex that is displaced toward the North Atlantic and associated with severe winter weather in the CEUS. The link between winter weather and these two stratospheric variations is seen in both composites of reanalysis near-surface temperatures and snow mass independently, using the newly developed severe weather index rAWSSI.

Our analysis suggests that reflection of stratospheric waves is an important element for the delivery of the CAOs to the CEUS during P3 events, similar to that found in Cohen *et al.* (*10*), in which a weakened and stretched lower SPV facilitates downstream stratospheric wave reflection that can lead to Arctic air advection in the lower tropospheric mid-latitudes. We note that similar stratospheric wave reflection also often precedes the start of P2 events and may act to precondition the northwest and central US with anomalously cold surface conditions, such that winter precipitation tends to fall as snow, particularly in the NWUS. However, the exact dynamical pathway by which the NWUS experiences extreme winter weather is not fully understood and requires further analyses.

The location of strongest winter cold anomalies has shifted from east of the Mississippi to west of the Mississippi in the US during the past decade, particularly for P2 and P3 days (Fig. 8), with an attendant increase in the ratio of P2 days to P3 days. An analysis of teleconnections reveals that P2 days occur more often during La Niña–like episodes (ONI index below zero), while P3 days tend to occur more often during El Niño–like episodes (ONI index above zero). On the basis of this, we hypothesize that a recent increase in La Niña events since 2000 coincides with recent increases in the ratio of P2 to P3 events, and the shifting of cold anomalies from east to west. It is not clear whether La Niña drives the P2 stratospheric variability directly or whether the stratospheric variability is a response to more complex tropospheric ENSO influences. Previously, it was found that under some warming greenhouse scenarios, the projected occurrence of consecutive La Niña winters increases, which can lead to more frequent global cold extremes, partly diminishing the overall warming of the globe (*42*).

Other teleconnections may also play a role in the stratospheric links to severe winter weather. For example, the QBO also shows a relationship to P2 and P3 frequency and may have a countering/enhancing effect on both stratospheric variations and subsequent surface temperatures. Here, P2 days tend to coincide with the westerly phase of the QBO, while P3 days tend to coincide with the easterly phase. However, this contrasts with Messori *et al.* (*29*) and Kretschmer *et al.* (*18*), who showed that the onsets of 33 of their 45 long-duration reflective events (bearing many similarities to our P3 days) coincided with the westerly phase of the QBO and 32 of the 45 events peaked during the westerly phase of the QBO. A complete understanding of the relationship between teleconnections and the stratospheric states requires an in-depth analysis of both tropospheric and stratospheric interactions, including three-dimensional WAF, eddy heights, and tropical Rossby wave connections (*29*, *30*).

This study has added to the steady growth in knowledge of how the polar stratosphere can influence mid-latitude winter weather including extreme weather. Specifically, it is one of the few studies to consider the stratosphere at two levels simultaneously and how variations in the shape of the SPV at various levels can inform surface weather. In terms of impacts, while most recent studies only consider surface temperature, this study extends extreme winter surface weather to include snow cover and snowfall. The recent shift in location of winter surface extremes from the CEUS to the NWUS and the concurrent increase in ENSO cold phases provide an intriguing insight into how variations in tropospheric/stratospheric interactions (including reflection) in conjunction with tropical influences can lead to different extreme winter weather impacts.

Our findings can be applied to improve subseasonal-to-seasonal forecasting. The polar vortex transition chart in Fig. 6 can guide forecasters in weeks 3 and 4 to anticipate periods of temperature transitions consistent with modes of the polar vortex. The placement and movement of temperature anomalies can be anticipated as shown in Fig. 8, on the basis of polar vortex strength. In addition, Figs. 8 and 9 can contribute to improved winter seasonal-mean outlooks by providing guidance where severe winter weather is more likely in the upcoming season. We note that NA winter-mean temperature anomalies for winter 2024/2025 were consistent with our analysis presented in Figs. 8E and 9.

## MATERIALS AND METHODS

### Experimental design

K-means clustering is used to separate the SPV into five variations based on upper-level (10 hPa) and lower-level (100 hPa) geopotential height anomalies. These clusters are examined for links to CONUS extreme cold and snow, including evidence of wave reflection and links to teleconnections.

### Reanalysis data

We use reanalysis output from NASA’s Modern Era Retrospective analysis for Research and Application, Version 2 [MERRA-2; (*43*–*45*)] for the period January to February 1980 to 2021. We consider January and February only because we are particularly interested in NA extreme winter surface conditions. The examined fields include pressure-level geopotential heights (dam), temperature (K), and winds (ms^−1^), along with single-level 2-m temperature (K) and snow mass (kg m^−2^). Poleward eddy heat flux V′T′ (K ms^−1^) at 100 hPa, where the primes indicate departures from the daily zonal means of meridional wind and temperature at each grid point, is used as a proxy for upward propagating waves into the stratosphere, as in Millin *et al.* (*30*). Anomalies are calculated by removing the long-term daily mean for each calendar day. WAF is calculated in accordance with Plumb’s (*46*) equation 7.1 and is subsequently filtered for the first three wave numbers. For WAF cross sections, the vertical and eastward filtered components are latitude-weighted and area-averaged over 55° to 75°N.

### Winter severity index

Winter severity is examined using the rAWSSI (*6*), a standardized daily measure of accumulated temperature, snowfall, and snow depth. The daily gridded values are computed for each grid point using European Centre for Medium-Range Weather Forecasts fifth-generation global analysis [ERA5; (*47*)], 2-m temperature, snowfall, and snow depth, according to the formulation for station-based AWSSI (*38*). The gridded rAWSSI dataset is validated by comparing the observed time series of AWSSI for major US cities (computed by the Midwest Regional Climate Center) with nearest-neighbor ERA5 grids (*6*). In the present analysis, these values are latitude-weighted and area-averaged over the CEUS (an area defined by 30° to 50°N and 104° to 70°W) and the NWUS (an area defined by 42° to 50°N and 130° to 104°W) to provide a daily index for each region, from 1980 to 2021. Separate indices are created for temperature, snowfall, and snow depth, as well as for the combined elements.

### Simple reflective index

The simple reflective index is similar to that used in (*22*, *28*, *29*), defined as the difference between the latitude-weighted area-mean 100-hPa standardized poleward eddy heat flux anomalies over northeastern Asia and Alaska/western Canada. Here, the northeastern Asia region is defined by 60° to 80°N and 120° to 180°E, and the Alaska/western Canada region is defined by 60° to 80°N and 180° to 240°E, chosen to best represent areas of anomalous wave activity for this study. We do not smooth the heat flux before standardizing as done in (*29*).

### Teleconnection indices

Teleconnections to large-scale circulations are examined using the daily AO index and the monthly QBO index from the National Oceanic and Atmospheric Administration (NOAA)’s Climate Prediction Center, the monthly ONI from NOAA’s Physical Science Laboratory, and the monthly PDO index from NOAA’s National Centers for Environmental Information.

### K-means method

Nonhierarchical K-means clustering (*48*) is used to separate daily January to February 1980 to 2021 60° to 90°N standardized geopotential height anomalies at 100 and 10 hPa into *K* nonoverlapping clusters, or representative patterns, through a process that iteratively assigns each day of data to a single cluster based on its similarity to the cluster centroid (effectively the composite of days assigned to the cluster). The algorithm is implemented using the Python sklearn.cluster KMeans package with init = k-means++, n_init = 50, max_iter = 500, and various values for *K*. As the first step in the process, anomalies are created by removing the long-term mean for each calendar day from each daily grid value and then standardizing at each grid point by dividing by the temporal standard deviation. The input data (both levels) are then weighted by the cosine of latitude and reduced using principal components analysis to maintain 95% of the variations. The number of clusters chosen is based on visual confirmation that the resulting clusters (i) are substantially different from each other throughout the depth of the stratosphere and (ii) show distinct differences in surface impacts for the CONUS. On the basis of this, we use a *K* = 5 solution (clusters P1 to P5), which is supported by both the Calinski-Harabasz and Silhouette scores from the Python’s sklearn package. We are particularly interested in clusters P2 and P3, which show extreme winter weather impacts in two different regions of the CONUS that are linked to two distinct variations of weakened lower stratospheric vortices and strong upper vortices. Several of our analyses consider cluster events, which are one or more consecutive days in a cluster, with each event identified by the cluster number, start date, and duration. Analyses that highlight P2 and P3 events use a minimum duration of 3 days for the events (e.g., Figs. 4, 5, and 8); all other analyses include the shorter 1- and 2-day events for each of the clusters. However, those shorter events comprise only 3.7% of the study period, and the results are largely insensitive to the inclusion of the shorter events.

The analysis was also performed using cluster sizes *K* = 6 and *K* = 7 (results shown in figs. S1 and S2, respectively). For each analysis, similar clusters to P2 and P3 occur (these are clusters P2K6 and P4K6, respectively, for *K* = 6 and clusters P3K7 and P5K7, respectively, for *K* = 7). We note that for the *K* = 6 solution, five of six clusters have similar counterparts to the *K* = 5 P1, P3, P4, and P5 clusters, with a new cluster (P2K6) composed of a subset of the days from the *K* = 5 P2 and P5 clusters. The *K* = 7 solution also contains clusters similar to the *K* = 5 P1, P3, P4, and P5 clusters, but with the two new clusters P2K7 and P4K7 composed of a subset of days from the *K* = 5 P2 cluster and the new cluster P3K7 composed of a subset of days from the *K* = 5 P2 and P5 clusters (similar to P2K6). For each solution, a single cluster is associated with anomalous cold in the NWUS, and a different single cluster is associated with anomalous cold in the CEUS. Thus, for these two types of clusters, our results are largely insensitive to our choice of *K* (that is, similar cold-CONUS clusters are revealed for *K* = 5, *K* = 6, and *K* = 7). In addition, the *K* = 6 and *K* = 7 solutions show that the clusters are not arbitrary: Extending to larger *K* results in the splitting of clusters into subclusters rather than creating new clusters, giving additional confidence in the *K* = 5 solution and its reproducibility. We have opted to use the smallest clustering (*K* = 5) that effectively showcases CONUS surface differences associated with stratospheric variations.

### Statistical analysis

Trends in cluster frequency are calculated using linear regression (Python scipy.stats package), using the Walt test with *t* distribution as the test statistic for the hypothesis test that the slope is zero. Statistical significance for all bar graphs is determined by bootstrapping, in which data (e.g., the rAWSSI index value assigned to each day) are randomly shuffled among the dates and sampled without replacement 1000 times, the results (i.e., the mean of the randomly assigned values per cluster) are sorted by value, and the lowest and highest 2.5% of the sorted values represent the 95% confidence interval of the mean value of the data. Values above or below the confidence interval are considered statistically significant departures from the mean. Statistical significance of map fields is similarly determined by bootstrapping; in this case, values at each grid box are randomly shuffled among the dates and sampled without replacement 1000 times as above to determine the 95% confidence interval of the mean.

## References

[1] IPCC, in *Climate Change 2023: Synthesis Report. Contribution of Working Groups I, II and III to the Sixth Assessment Report of the Intergovernmental Panel on Climate Change*, Core Writing Team, H. Lee, and J. Romero, Eds. (IPCC, Geneva, Switzerland, 2023) pp. 35–115. doi: 10.59327/IPCC/AR6-9789291691647.

[2] M. Rantanen, A. Y. Karpechko, A. Lipponen, K. Nordling, P. Hyvärinen, K. Ruosteenoja, T. Vihma, A. Laaksonen, The Arctic has warmed nearly four times faster than the globe since 1979. Commun. Earth Environ. 3, 168 (2022).

[3] J. Cohen, L. Agel, M. Barlow, C. I. Garfinkel, I. White, Response to limited surface impacts of the January 2021 sudden stratospheric warming. Nat. Commun. 14, 3289 (2023). 37286575 10.1038/s41467-023-38772-3 PMC10247762

[4] D. Singh, Y. S. Bekris, C. D. W. Rogers, J. Doss-Gollin, E. D. Coffel, D. A. Kalashnikov, Enhanced solar and wind potential during widespread temperature extremes across the U.S. interconnected energy grids. Environ. Res. Lett. 19, 044018 (2024).

[5] R. Blackport, J. C. Fyfe, Amplified warming of North American cold extremes linked to human-induced changes in temperature variability. Nat. Commun. 15, 5864 (2024). 38997288 10.1038/s41467-024-49734-8 PMC11245492

[6] J. Cohen, J. A. Francis, K. Pfeiffer, Anomalous Arctic warming linked with severe winter weather in Northern Hemisphere continents. Commun. Earth Environ. 5, 557 (2024).

[7] J. Cohen, J. Foster, M. Barlow, K. Saito, J. Jones, Winter 2009–2010: A case study of an extreme Arctic Oscillation event. Geophys. Res. Lett. 37, L17707 (2010).

[8] J. Cohen, L. Agel, M. Barlow, J. C. Furtado, M. Kretschmer, V. Wendt, The “Polar Vortex” winter of 2013/2014. J. Geophys. Res. Atmos. 127, e2022JD036493 (2022).

[9] Washington Post Capitol Weather Gang, “Polar vortex brings more historic cold in eastern US” (2015); https://washingtonpost.com/news/capital-weather-gang/wp/2015/02/19/arctic-outbreak-shatters-records-in-eastern-u-s-coldest-yet-to-come/.

[10] J. Cohen, L. Agel, M. Barlow, C. I. Garfinkel, I. White, Linking Arctic variability and change with extreme winter weather in the United States. Science 373, 1116–1121 (2021). 34516838 10.1126/science.abi9167

[11] National Centers for Environmental Information, “Monthly Climate Reports” (2025); https://ncei.noaa.gov/access/monitoring/monthly-report/national/202501.

[12] M. P. Baldwin, T. J. Dunkerton, Propagation of the Arctic Oscillation from the stratosphere to the troposphere. J. Geophys. Res. 104, 30937–30946 (1999).

[13] A. J. Charlton, L. M. Polvani, A new look at stratospheric sudden warmings. Part I: Climatology and modeling benchmarks. J. Climate 20, 449–469 (2007).

[14] M. P. Baldwin, T. J. Dunkerton, Stratospheric harbingers of anomalous weather regimes. Science 294, 581–584 (2001). 11641495 10.1126/science.1063315

[15] E. W. Kolstad, T. Breiteig, A. A. Scaife, The association between stratospheric weak polar vortex events and cold air outbreaks in the Northern Hemisphere. Q.J.R. Meteorol. Soc. 136, 886–893 (2010).

[16] J. Cohen, M. Barlow, P. J. Kushner, K. Saito, Stratosphere-troposphere coupling and links with Eurasian land-surface variability. J. Climate 20, 5335–5343 (2007).

[17] D. W. J. Thompson, J. M. Wallace, Regional climate impacts of the Northern Hemisphere annular mode. Science 293, 85–89 (2001). 11441178 10.1126/science.1058958

[18] M. Kretschmer, J. Cohen, V. Matthias, J. Runge, D. Coumou, The different stratospheric influence on cold-extremes in Eurasia and North America. npj Clim. Atmos. Sci. 1, 44 (2018).

[19] J. Perlwitz, N. Harnik, Observational evidence of a stratospheric influence on the troposphere by planetary wave reflection. J. Climate 16, 3011–3026 (2003).

[20] J. Perlwitz, N. Harnik, Downward coupling between the stratosphere and troposphere: The relative roles of wave and zonal mean processes. J. Climate 17, 4902–4909 (2004).

[21] K. Kodera, H. Mukougawa, S. Itoh, Tropospheric impact of reflected planetary waves from the stratosphere. Geophys. Res. Lett. 35, L16806 (2008).

[22] V. Matthias, M. Kretschmer, The influence of stratospheric wave reflection on North American cold spells. Mon. Weather Rev. 148, 1675–1690 (2020).

[23] X. Shen, L. Wang, A. A. Scaife, S. C. Hardiman, P. Xu, The stratosphere–troposphere oscillation as the dominant intraseasonal coupling mode between the stratosphere and troposphere. J. Climate 36, 2259–2276 (2023).

[24] I. Weinberger, C. I. Garfinkel, N. Harnik, N. Paldor, Transmission and reflection of upward-propagating Rossby waves in the lowermost stratosphere: Importance of the tropopause inversion layer. J. Atmos. Sci. 79, 3263–3274 (2022).

[25] K. Kodera, H. Mukougawa, A. Fujii, Influence of the vertical and zonal propagation of stratospheric planetary waves on tropospheric blockings. J. Geophys. Res. Atmos. 118, 8333–8345 (2013).

[26] K. Kodera, H. Mukougawa, P. Maury, M. Ueda, C. Claud, Absorbing and reflecting sudden stratospheric warming events and their relationship with tropospheric circulation. J. Geophys. Res. Atmos. 12, 80–94 (2016).

[27] D. Nath, W. Chen, L. Wang, Y. Ma, Planetary wave reflection and its impact on tropospheric cold weather over Asia during January 2008. Adv. Atmos. Sci. 31, 851–862 (2014).

[28] M. Kretschmer, D. Coumou, L. Agel, M. Barlow, E. Tziperman, J. Cohen, More-persistent weak stratospheric polar vortex states linked to cold extremes. Bull. Am. Meteorol. Soc. 99, 49–60 (2018).

[29] G. Messori, M. Kretschmer, S. H. Lee, V. Wendt, Stratospheric downward wave reflection events modulate North American weather regimes and cold spells. Weather Clim. Dynam. 3, 1215–1236 (2022).

[30] O. T. Millin, J. C. Furtado, J. B. Basara, Characteristics, evolution, and formation of cold air outbreaks in the great plains of the United States. J. Climate 35, 4585–4602 (2022).

[31] X. Ding, G. Chen, L. Sun, P. Zhang, Distinct North American cooling signatures following the zonally symmetric and asymmetric modes of winter stratospheric variability. Geophys. Res. Lett. 49, e2021GL096076 (2022).

[32] X. Ding, G. Chen, W. Ma, Stratosphere-troposphere coupling of Extreme Stratospheric Wave activity in CMIP6 models. J. Geophys. Res. Atmos. 128, e2023JD038811 (2023).

[33] X. Ding, G. Chen, P. Zhang, D. I. V. Domeisen, C. Orbe, Extreme stratospheric wave activity as harbingers of cold events over North America. Commun. Earth Environ. 4, 187 (2023). 38665179 10.1038/s43247-023-00845-y PMC11041758

[34] A. Butler, J. Sjoberg, D. Seidel, NOAA ESRL Chemical Science Division, Sudden Stratospheric Warming Compendium, Version 1.0 (NOAA National Centers for Environmental Information [NCEI], 2016); 10.7289/V5NS0RWP.

[35] S. H. Lee, J. C. Furtado, A. J. Charlton-Perez, Wintertime North American weather regimes and the Arctic stratospheric polar vortex. Geophys. Res. Lett. 46, 14892–14900 (2019).

[36] S. H. Lee, G. Messori, The dynamical footprint of year-round North American weather regimes. Geophys. Res. Lett. 51, e2023GL107161 (2024).

[37] O. T. Millin, J. C. Furtado, C. Malloy, The impact of North American winter weather regimes on electricity load in the central United States. npj Clim. Atmos. Sci. 7, 254 (2024).

[38] B. E. Mayes Boustead, S. D. Hilberg, M. D. Shulski, M. D. Hubbard, The Accumulated Winter Season Severity Index (AWSSI). J. Appl. Meteorol. Climatol. 54, 1693–1712 (2015).

[39] C. I. Garfinkel, I. Weinberger, I. P. White, L. D. Oman, V. Aquila, Y.-K. Lim, The salience of nonlinearities in the boreal winter response to ENSO: North Pacific and North America. Climate Dynam. 52, 4429–4446 (2019). 10.1007/s00382-019-04805-1 PMC6769094 31631950

[40] C. I. Garfinkel, C. Schwartz, A. H. Butler, D. Domeisen IV, S.-K. Seok-Woo Son, I. P. White, Weakening of the teleconnection from El Niño–Southern Oscillation to the Arctic stratosphere over the past few decades: What can be learned from subseasonal forecast models? J. Geophys. Res. Atmos. 124, 7683–7696 (2019).

[41] D. Domeisen IV, C. I. Garfinkel, A. H. Butler, The teleconnection of El Niño Southern Oscillation to the stratosphere. Rev. Geophys. 57, 5–47 (2019).

[42] T. Geng, F. Jia, W. Cai, L. Wu, B. Gan, Z. Jing, S. Li, M. J. McPhaden, Increased occurrences of consecutive La Niña events under global warming. Nature 619, 774–781 (2023). 37495880 10.1038/s41586-023-06236-9 PMC10371868

[43] GMAO, *MERRA-2 inst3_3d_asm_Np: 3d, 3-Hourly, Instantaneous, Pressure-Level, Assimilation, Assimilated Meteorological Fields, version 5.12.4* (Goddard Earth Sciences Data and Information Services Center, 2015).

[44] GMAO, *MERRA-2 tavg1_2d_lnd_Nx: 2d, 1-Hourly, Time-Averaged, Single-Level, Assimilation, Land Surface Diagnostics, version 5.12.4* (Goddard Earth Sciences Data and Information Services Center, 2015).

[45] GMAO, *MERRA-2 tavg1_2d_slv_Nx: 2d, 1-Hourly, Time-Averaged, Single-Level, Assimilation, Single-Level Diagnostics, version 5.12.4* (Goddard Earth Sciences Data and Information Services Center, 2015).

[46] R. A. Plumb, On the three-dimensional propagation of stationary waves. J. Atmos. Sci. 42, 217–229 (1985).

[47] H. Hersbach, B. Bell, P. Berrisford, G. Biavati, A. Horányi, J. Muñoz Sabater, J. Nicolas, C. Peubey, R. Radu, I. Rozum, D. Schepers, A. Simmons, C. Soci, D. Dee, J-N Thépaut, ERA5 hourly data on single levels from 1940 to present, Copernicus Climate Change Service (C3S) Climate Data Store (CDS) (2023).

[48] E. Diday, J. C. Simon, “Clustering analysis,” in *Digital Pattern Recognition*, K. S. Fu, Ed. (Springer Berlin Heidelberg, 1976), pp. 47–94.

