# Supplementary material for: Cold-air outbreaks in the continental US: Connections with stratospheric variations
Source: Sci Adv. 2025 Jul 11;11(28):eadq9557. doi: 10.1126/sciadv.adq9557 (PMC12248286; doi:10.1126/sciadv.adq9557)
Supplement: Supplementary file 1 — Figs. S1 and S2 [file sciadv.adq9557_sm.pdf]

Science Advances
AAAS

Supplementary Materials for

Cold-air outbreaks in the continental US: Connections with stratospheric variations

Laurie Agel *et al.*

Corresponding author: Laurie Agel, laurie_agel@uml.edu

Sci. Adv. **11**, eadq9557 (2025)
DOI: 10.1126/sciadv.adq9557

This PDF file includes:

Figs. S1 and S2

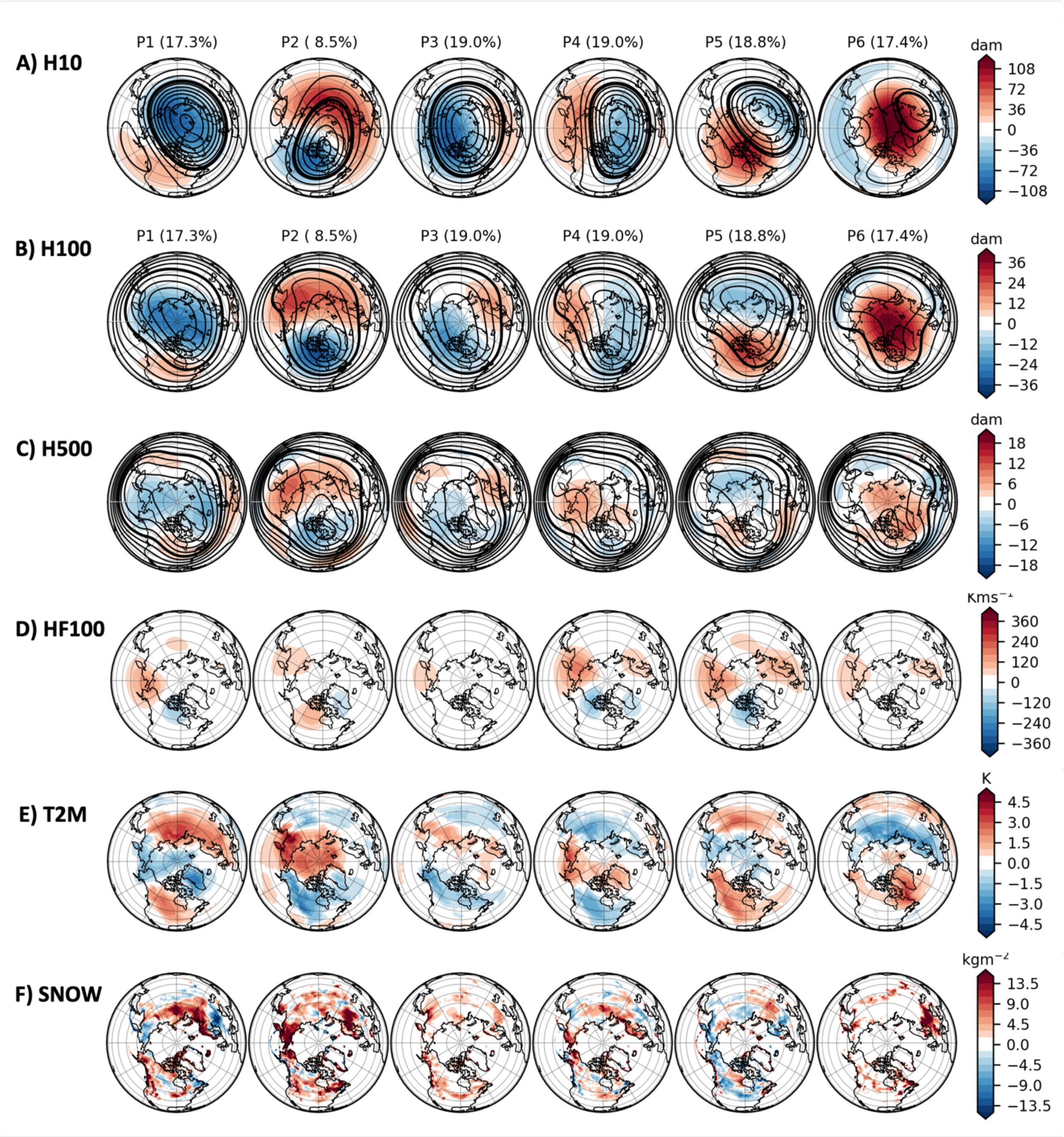

Fig. S1. Clustering results as in Figure 1 and 2, but for k=6 clusters. MERRA-2 composite (A) 10-hPa geopotential heights (contours in 3-dam intervals, thick contour for 300 dam) and anomalies (dam, shading), (B) 100-hPa geopotential heights (contours in 2-dam intervals, thick contour for 158 dam) and anomalies (dam, shading), (C) 500-hPa geopotential heights (dam, contours with thick contour for 540 dam, and anomalies shaded), (D) 100-hPa heat flux anomalies (K ms^{-1}, shaded), (E) 2-m temperature anomalies (K, shaded), and (F) surface snow mass anomalies (kg m^{-2}, shaded) for clusters labeled P1–P6.

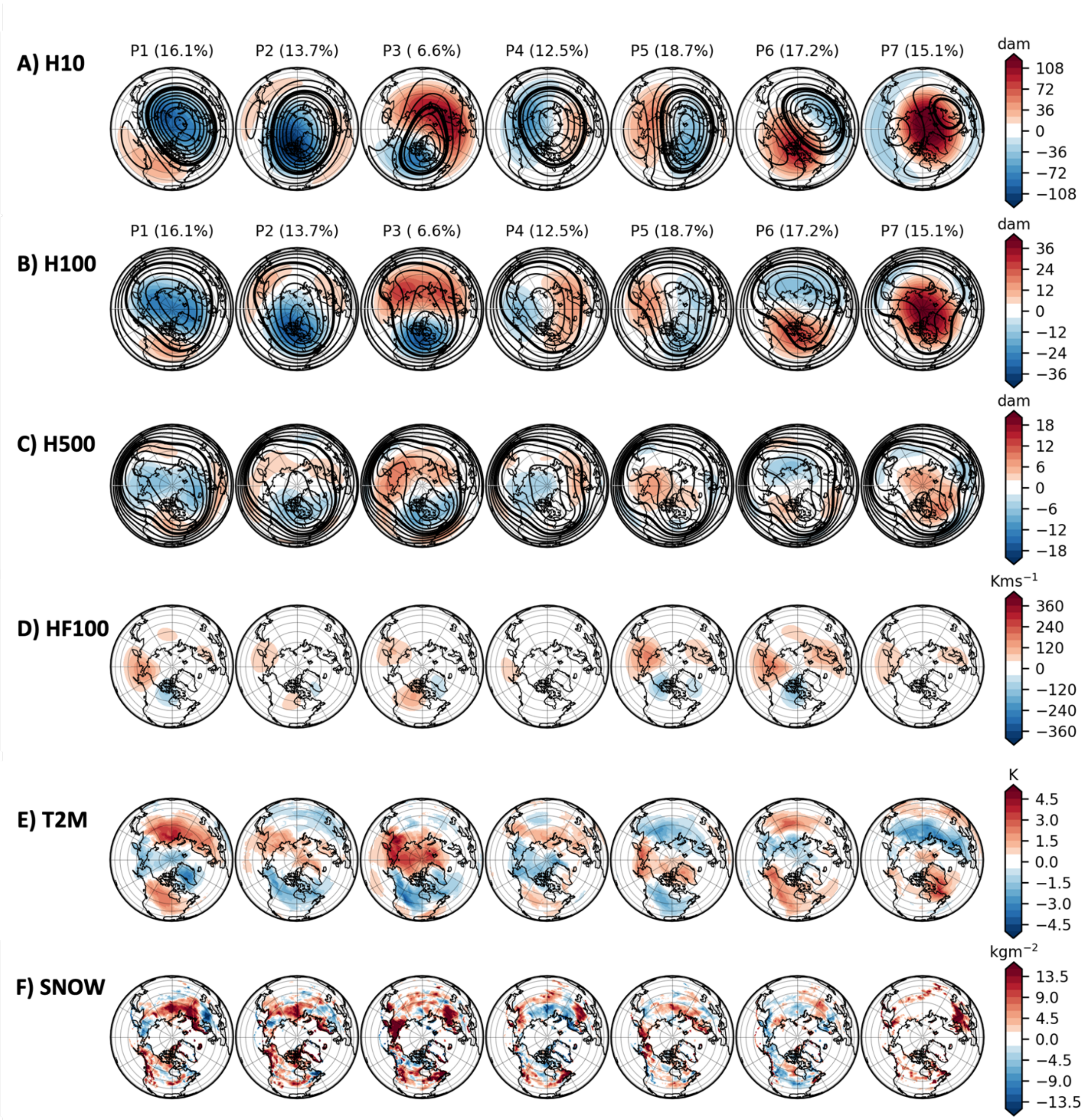

Fig. S2. Clustering results as in Figure 1 and 2, but for k=7 clusters. MERRA-2 composite **(A)** 10-hPa geopotential heights (contours in 3-dam intervals, thick contour for 300 dam) and anomalies (dam, shading), **(B)** 100-hPa geopotential heights (contours in 2-dam intervals, thick contour for 158 dam) and anomalies (dam, shading), **(C)** 500-hPa geopotential heights (dam, contours with thick contour for 540 dam, and anomalies shaded), **(D)** 100-hPa heat flux anomalies (K ms^{-1}, shaded), **(E)** 2-m temperature anomalies (K, shaded), and **(F)** surface snow mass anomalies (kg m^{-2}, shaded) for clusters labeled P1–P6.
